# Ca_5_Zr_3_F_22_

**DOI:** 10.1107/S1600536812008495

**Published:** 2012-03-03

**Authors:** Abdelghani Oudahmane, Malika El-Ghozzi, Daniel Avignant

**Affiliations:** a Clermont Université, Université Blaise Pascal, Institut de Chimie de Clermont-Ferrand, BP 10448, 63000 Clermont-Ferrand, France and CNRS, UMR 6296, ICCF, BP 80026, 63171 Aubière, France

## Abstract

Single crystals of Ca_5_Zr_3_F_22_, penta­calcium trizirconium docosafluoride, were obtained unexpectedly by solid-state reaction between CaF_2_ and ZrF_4_ in the presence of AgF. The structure of the title compound is isotypic with that of Sr_5_Zr_3_F_22_ and can be described as being composed of layers with composition [Zr_3_F_20_]^8−^ made up from two different [ZrF_8_]^4−^ square anti­prisms (one with site symmetry 2) by corner-sharing. The layers extending parallel to the (001) plane are further linked by Ca^2+^ cations, forming a three-dimensional network. Amongst the four crystallographically different Ca^2+^ ions, three are located on twofold rotation axes. The Ca^2+^ ions exhibit coordination numbers ranging from 8 to 12, depending on the cut off, with very distorted fluorine environments. Two of the Ca^2+^ ions occupy inter­stices between the layers whereas the other two are located in void spaces of the [Zr_3_F_20_]^8−^ layer and alternate with the two Zr atoms along [010]. The crystal under investigation was an inversion twin.

## Related literature

For the isotypic Sr analogue, see: Le Bail (1996 ▶). The crystal chemistry of fluorides has been reviewed by Babel & Tressaud (1985 ▶). For phase relationships in the CaF_2_–ZrF_4_ system, see: L’Helgoualch *et al.* (1971 ▶); Kotsar *et al.* (1973 ▶); Ratnikov *et al.* (1977 ▶); Laval *et al.* (1987 ▶). For bond-valence analysis, see: Brese & O’Keeffe (1991 ▶).

## Experimental

### 

#### Crystal data

Ca_5_Zr_3_F_22_
*M* *_r_* = 892.06
Orthorhombic, 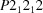

*a* = 9.9844 (3) Å
*b* = 7.4059 (2) Å
*c* = 9.9046 (3) Å
*V* = 732.38 (4) Å^3^
*Z* = 2
Mo *K*α radiation
μ = 4.09 mm^−1^
*T* = 296 K
0.19 × 0.06 × 0.03 mm

#### Data collection

Bruker APEXII CCD diffractometer
Absorption correction: multi-scan (*SADABS*; Bruker, 2008 ▶) *T*_min_ = 0.587, *T*_max_ = 0.747
7993 measured reflections
3466 independent reflections
2909 reflections with *I* > 2σ(*I*)
*R*_int_ = 0.037

#### Refinement

*R*[*F* ^2^ > 2σ(*F* ^2^)] = 0.036
*wR*(*F* ^2^) = 0.058
*S* = 0.99
3466 reflections
139 parameters
Δρ_max_ = 0.88 e Å^−3^
Δρ_min_ = −0.77 e Å^−3^
Absolute structure: Flack (1983 ▶), 1462 Friedel pairs
Flack parameter: 0.0 (4)

### 

Data collection: *APEX2* (Bruker, 2008 ▶); cell refinement: *SAINT* (Bruker, 2008 ▶); data reduction: *SAINT*; program(s) used to solve structure: *SHELXS97* (Sheldrick, 2008 ▶); program(s) used to refine structure: *SHELXL97* (Sheldrick, 2008 ▶); molecular graphics: *CaRine* (Boudias & Monceau, 1998 ▶) and *ORTEP-3 for Windows* (Farrugia, 1997 ▶); software used to prepare material for publication: *SHELXTL* (Sheldrick, 2008 ▶).

## Supplementary Material

Crystal structure: contains datablock(s) I, global. DOI: 10.1107/S1600536812008495/wm2595sup1.cif

Structure factors: contains datablock(s) I. DOI: 10.1107/S1600536812008495/wm2595Isup2.hkl

Additional supplementary materials:  crystallographic information; 3D view; checkCIF report

## Figures and Tables

**Fig. 1.**
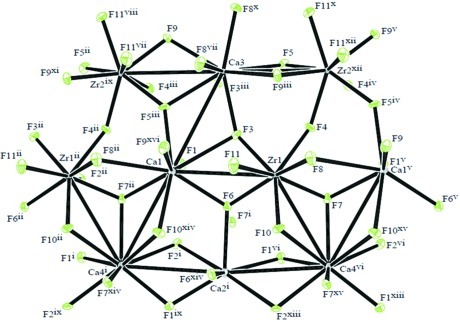
View of the polyhedral linkage in Ca5Zr3F22. Displacement ellipsoids are drawn at the 50% probability level. Symmetry codes: (i) 3/2 - x, -1/2 + y, 2 - z; (ii) x, -1 + y, z; (iii) 2 - x, -y, z; (iv) 2 - x, 1 - y, z; (v) x, 1 + y, z; (vi) 3/2 - x, 1/2 + y, 2 - z; (vii) 3/2 - x, -1/2 + y, 1 - z; (viii) 1/2 + x, -1/2 - y, 1 - z; (ix) -1/2 + x, -1/2 - y, 2 - z; (x) 1/2 + x, 1/2 - y, 1 - z; (xi) 2 - x, -1 - y, z; (xii) 3/2 - x, 1/2 + y, 1 - z; (xiii) -1/2 + x, 1/2 - y, 2 - z; (xiv) 1 - x, -y, z; (xv) 1 - x, 1 - y, z; (xvi) -1/2 + x, -1/2 - y, 1 - z;

**Fig. 2.**
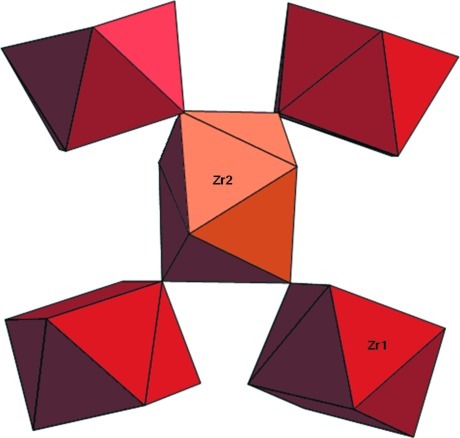
Details of the connection between Zr2 and Zr1 square antiprisms in Ca5Zr3F22.

**Fig. 3.**
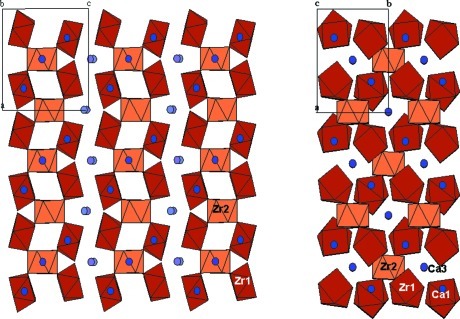
Projection of the structure of Ca5Zr3F22 along [010] showing the [Zr3F20]8- layers held together by Ca2+ ions.

**Fig. 4.**
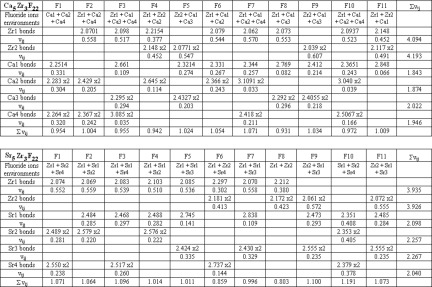
Bond-valence analysis of the structures of Ca5Zr3F22 (top) and Sr5Zr3F22 (bottom) using parameters for solids (RZr—F = 1.854, RCa—F = 1.842, RSr—F = 2.019) from Brese & O'Keeffe (1991). It should be noted that the atom labelling in the two isotypic structures is different.

## supplementary crystallographic information

### Comment

Despite several reports related to investigations of the pseudo-binary system CaF_2_–ZrF_4_ (L'Helgoualch *et al.* 1971; Kotsar *et al.* 1973; Ratnikov *et al.* 1977; Babel & Tressaud, 1985; Laval *et al.* 1987) the title compound has never been mentioned previously. Therefore the determination of its crystal structure was of prime importance to complete a thorough examination of the phase diagram of this system.

The orthorhombic structure of the title compound is isotypic with that of Sr_5_Zr_3_F_22_ reported by Le Bail (1996). As with this latter, the structure of Ca_5_Zr_3_F_22_ has also been determined from an inversion twinned crystal although the crystals were grown in very different ways. Figure 1 displays the polyhedral string in Ca_5_Zr_3_F_22_. Both Zr1 and Zr2 cations are 8-coordinated by the fluoride ions, in distorted square antiprismatic environments. The square antiprism surrounding Zr2 (site symmetry 2) with Zr—F distances ranging from 2.0771 (19) Å to 2.148 (2) Å is less distorted than that surrounding Zr1 (site symmetry 1) where Zr—F distances range from 2.062 (2) to 2.2154 (19) Å. Each Zr2 square antiprism is connected by sharing corners to four Zr1 square antiprisms as shown in Fig. 2. The crystal structure is built up from layers of corner-sharing [ZrF_8_]^4-^ square antiprisms. The layers extending parallel to (001) are further held together by Ca^2+^ cations to form the three-dimensional network (Fig. 3). The calcium ions are divided into four crystallographically different atoms exhibiting coordination numbers from 8 to 12, depending on the cut off, with very distorted fluorine environments. The bond-valence analysis of the title structure carried out using Brese & O'Keeffe's R_ij_ parameters for solids (Brese & O'Keeffe, 1991) is displayed in Figure 4. Large deviations from the ideal value 2 have been obtained for both Ca1 and Ca2. For this latter the value of 1.730 valence units (v.u.) is obtained with Ca—F distances up to 2.661 Å considered as relevant for the first coordination sphere whereas the value of 1.843 v.u. is reached with four additional interactions involving distances up to 3.109 Å. Despite these four additional contributions the formal charge for Ca2 still shows a deficit. For comparison, bond-valence calculations have also been performed for the homologuous Sr_5_Zr_3_F_22_ from bond lengths reported by Le Bail (1996). Significant deviations (see Fig. 4) are also observed for the alkaline earth cations but the formal charges are in excess in this case. This may be most likely related to the size difference of the ionic radii of Ca^2+^ and Sr^2+^. Ca2 and Ca4 occupy interstices between the layers whereas Ca3 and Ca1 are located in void spaces of the [Zr_3_F_20_]^8-^ layer and alternate with Zr2 and Zr1, respectively, along [010]. Thus Ca3 and Ca4 also appear as being located in channels parallel to [001].

### Experimental

Single crystals of Ca_5_Zr_3_F_22_ were unexpectedly obtained from an equimolar mixture of AgF, CaF_2_ and ZrF_4_ heated at 873 K in a sealed platinum tube during the study of the phase diagram of the ternary system AgF–CaF_2_–ZrF_4_. After heating at this temperature for 24 h, the sample was cooled to room temperature at the rate of 5 K^.^h^-1^ for the first 24 h and then by switching the power off. Small platelet like crystals of Ca_5_Zr_3_F_22_ could have been extracted from the batch.

Both AgF and CaF_2_ were commercial products whereas ZrF_4_ was prepared by direct fluorination of ZrO_2_ under pure fluorine gas flow at 873 K with intermediate grindings. CaF_2_ was also heated at 873 K overnight under fluorine gas flow prior to use.

### Refinement

The highest residual peak in the final difference Fourier map was located 0.04 Å from atom Zr2 and the deepest hole was located 1.05 Å from atom F9.

### Crystal data

**Table:**

Ca_5_Zr_3_F_22_	*F*(000) = 836
*M**_r_* = 892.06	*D*_x_ = 4.045 Mg m^−^^3^
Orthorhombic, *P*2_1_2_1_2	Mo *K*α radiation, λ = 0.71073 Å
Hall symbol: P 2 2ab	Cell parameters from 1507 reflections
*a* = 9.9844 (3) Å	θ = 4.0–35.8°
*b* = 7.4059 (2) Å	µ = 4.09 mm^−^^1^
*c* = 9.9046 (3) Å	*T* = 296 K
*V* = 732.38 (4) Å^3^	Platelet, colourless
*Z* = 2	0.19 × 0.06 × 0.03 mm

### Data collection

**Table:**

Bruker APEXII CCD diffractometer	3466 independent reflections
Radiation source: fine-focus sealed tube	2909 reflections with *I* > 2σ(*I*)
Graphite monochromator	*R*_int_ = 0.037
Detector resolution: 8.3333 pixels mm^-1^	θ_max_ = 36.0°, θ_min_ = 3.4°
ω and φ–scans	*h* = −16→16
Absorption correction: multi-scan (*SADABS*; Bruker, 2008)	*k* = −12→6
*T*_min_ = 0.587, *T*_max_ = 0.747	*l* = −16→12
7993 measured reflections	

### Refinement

**Table:**

Refinement on *F*^2^	Primary atom site location: structure-invariant direct methods
Least-squares matrix: full	Secondary atom site location: difference Fourier map
*R*[*F*^2^ > 2σ(*F*^2^)] = 0.036	*w* = 1/[σ^2^(*F*_o_^2^) + (0.0112*P*)^2^] where *P* = (*F*_o_^2^ + 2*F*_c_^2^)/3
*wR*(*F*^2^) = 0.058	(Δ/σ)_max_ = 0.001
*S* = 0.99	Δρ_max_ = 0.88 e Å^−^^3^
3466 reflections	Δρ_min_ = −0.77 e Å^−^^3^
139 parameters	Absolute structure: Flack (1983), 1462 Friedel pairs
0 restraints	Flack parameter: 0.0 (4)

### Special details

**Table:**

Geometry. All e.s.d.'s (except the e.s.d. in the dihedral angle between two l.s. planes) are estimated using the full covariance matrix. The cell e.s.d.'s are taken into account individually in the estimation of e.s.d.'s in distances, angles and torsion angles; correlations between e.s.d.'s in cell parameters are only used when they are defined by crystal symmetry. An approximate (isotropic) treatment of cell e.s.d.'s is used for estimating e.s.d.'s involving l.s. planes.
Refinement. Refinement of *F*^2^ against ALL reflections. The weighted *R*-factor *wR* and goodness of fit *S* are based on *F*^2^, conventional *R*-factors *R* are based on *F*, with *F* set to zero for negative *F*^2^. The threshold expression of *F*^2^ > σ(*F*^2^) is used only for calculating *R*-factors(gt) *etc*. and is not relevant to the choice of reflections for refinement. *R*-factors based on *F*^2^ are statistically about twice as large as those based on *F*, and *R*- factors based on ALL data will be even larger.

### Fractional atomic coordinates and isotropic or equivalent isotropic displacement parameters (Å^2^)

**Table:**

	*x*	*y*	*z*	*U*_iso_*/*U*_eq_	
Zr1	0.71210 (3)	0.25190 (5)	0.77653 (3)	0.00516 (5)	
Zr2	0.5000	0.0000	0.46134 (5)	0.00610 (9)	
Ca1	0.71132 (6)	−0.23978 (10)	0.75189 (6)	0.00980 (11)	
Ca2	1.0000	0.5000	0.94489 (11)	0.01098 (18)	
Ca3	1.0000	0.0000	0.53761 (10)	0.00549 (16)	
Ca4	1.0000	0.0000	0.97774 (11)	0.01109 (19)	
F1	0.88717 (17)	−0.2405 (3)	0.89417 (19)	0.0127 (4)	
F2	0.85135 (16)	0.2410 (3)	0.93117 (18)	0.0108 (3)	
F3	0.8514 (2)	0.0603 (2)	0.7085 (3)	0.0170 (5)	
F4	0.88835 (19)	0.4117 (2)	0.7118 (3)	0.0126 (4)	
F5	1.10993 (18)	0.2790 (3)	0.6049 (2)	0.0128 (4)	
F6	0.65761 (18)	0.0167 (3)	0.8771 (2)	0.0113 (4)	
F7	0.69569 (18)	0.4936 (3)	0.87846 (19)	0.0114 (4)	
F8	0.6504 (2)	0.4338 (3)	0.6301 (2)	0.0156 (5)	
F9	1.10631 (18)	−0.2758 (3)	0.4672 (2)	0.0131 (4)	
F10	0.51040 (18)	0.2795 (2)	0.83055 (19)	0.0139 (4)	
F11	0.6171 (2)	0.0833 (3)	0.6283 (3)	0.0177 (5)	

### Atomic displacement parameters (Å^2^)

**Table:**

	*U*^11^	*U*^22^	*U*^33^	*U*^12^	*U*^13^	*U*^23^
Zr1	0.00491 (11)	0.00437 (10)	0.00621 (12)	0.00005 (14)	−0.00025 (10)	0.00010 (14)
Zr2	0.0070 (2)	0.00559 (19)	0.0057 (2)	−0.0005 (2)	0.000	0.000
Ca1	0.0079 (2)	0.0118 (3)	0.0097 (3)	−0.0004 (3)	0.0000 (2)	0.0002 (3)
Ca2	0.0104 (4)	0.0085 (4)	0.0139 (5)	0.0006 (4)	0.000	0.000
Ca3	0.0047 (4)	0.0059 (4)	0.0059 (4)	0.0005 (5)	0.000	0.000
Ca4	0.0097 (4)	0.0065 (4)	0.0170 (5)	−0.0010 (4)	0.000	0.000
F1	0.0108 (8)	0.0113 (8)	0.0161 (9)	−0.0008 (9)	−0.0033 (7)	−0.0009 (10)
F2	0.0100 (8)	0.0125 (8)	0.0098 (8)	−0.0027 (10)	−0.0044 (6)	0.0024 (10)
F3	0.0194 (11)	0.0113 (9)	0.0203 (13)	0.0026 (8)	0.0086 (10)	−0.0019 (9)
F4	0.0118 (10)	0.0128 (9)	0.0133 (11)	−0.0041 (7)	0.0021 (9)	0.0004 (8)
F5	0.0103 (8)	0.0159 (11)	0.0122 (10)	0.0038 (7)	−0.0044 (7)	−0.0006 (8)
F6	0.0129 (9)	0.0090 (9)	0.0120 (10)	−0.0019 (8)	0.0002 (7)	−0.0007 (8)
F7	0.0129 (9)	0.0084 (8)	0.0130 (10)	−0.0016 (9)	−0.0016 (8)	−0.0023 (9)
F8	0.0202 (12)	0.0145 (10)	0.0120 (12)	0.0008 (8)	−0.0061 (9)	0.0047 (8)
F9	0.0135 (9)	0.0108 (10)	0.0151 (10)	−0.0034 (8)	0.0041 (7)	0.0002 (8)
F10	0.0077 (8)	0.0177 (10)	0.0161 (10)	0.0009 (8)	0.0012 (8)	−0.0044 (7)
F11	0.0233 (12)	0.0146 (10)	0.0151 (13)	−0.0028 (9)	−0.0065 (10)	−0.0028 (9)

### Geometric parameters (Å, º)

**Table:**

Zr1—F7	2.062 (2)	Ca2—F6^x^	2.366 (2)
Zr1—F2	2.0701 (16)	Ca2—F2^xi^	2.429 (2)
Zr1—F8	2.073 (2)	Ca2—F2	2.429 (2)
Zr1—F6	2.079 (2)	Ca2—F4	2.645 (3)
Zr1—F10	2.0937 (18)	Ca2—F4^xi^	2.645 (3)
Zr1—F3	2.098 (2)	Ca2—F10^x^	3.040 (2)
Zr1—F11	2.148 (2)	Ca2—F10^ix^	3.040 (2)
Zr1—F4	2.2154 (19)	Ca2—F7	3.1091 (18)
Zr2—F5^i^	2.0771 (19)	Ca2—F7^xi^	3.1091 (18)
Zr2—F5^ii^	2.0771 (19)	Ca3—F8^i^	2.292 (2)
Zr2—F9^iii^	2.0939 (18)	Ca3—F8^xii^	2.292 (2)
Zr2—F9^iv^	2.0939 (18)	Ca3—F3^vi^	2.295 (2)
Zr2—F11	2.117 (2)	Ca3—F3	2.295 (2)
Zr2—F11^v^	2.117 (2)	Ca3—F9	2.4055 (19)
Zr2—F4^ii^	2.148 (2)	Ca3—F9^vi^	2.4055 (19)
Zr2—F4^i^	2.148 (2)	Ca3—F5	2.4327 (19)
Ca1—F1	2.2514 (18)	Ca3—F5^vi^	2.4327 (19)
Ca1—F5^vi^	2.3214 (19)	Ca4—F1	2.264 (2)
Ca1—F6	2.331 (2)	Ca4—F1^vi^	2.264 (2)
Ca1—F7^vii^	2.344 (2)	Ca4—F2^vi^	2.367 (2)
Ca1—F10^v^	2.3651 (19)	Ca4—F2	2.367 (2)
Ca1—F9^iv^	2.412 (2)	Ca4—F7^ix^	2.418 (2)
Ca1—F3	2.661 (2)	Ca4—F7^xiii^	2.418 (2)
Ca1—F8^vii^	2.769 (2)	Ca4—F10^ix^	2.5067 (19)
Ca1—F11	2.848 (2)	Ca4—F10^xiii^	2.5067 (19)
Ca2—F1^viii^	2.283 (2)	Ca4—F3	3.085 (3)
Ca2—F1^vi^	2.283 (2)	Ca4—F3^vi^	3.085 (3)
Ca2—F6^ix^	2.366 (2)		
			
F7—Zr1—F2	74.04 (8)	F1^viii^—Ca2—F7	58.94 (6)
F7—Zr1—F8	75.80 (8)	F1^vi^—Ca2—F7	115.01 (6)
F2—Zr1—F8	137.96 (9)	F6^ix^—Ca2—F7	143.78 (7)
F7—Zr1—F6	118.16 (7)	F6^x^—Ca2—F7	60.56 (6)
F2—Zr1—F6	77.79 (8)	F2^xi^—Ca2—F7	126.67 (6)
F8—Zr1—F6	143.31 (8)	F2—Ca2—F7	51.62 (6)
F7—Zr1—F10	73.37 (7)	F4—Ca2—F7	53.12 (6)
F2—Zr1—F10	117.44 (7)	F4^xi^—Ca2—F7	103.35 (6)
F8—Zr1—F10	80.18 (8)	F10^x^—Ca2—F7	97.57 (5)
F6—Zr1—F10	73.00 (7)	F10^ix^—Ca2—F7	100.25 (5)
F7—Zr1—F3	142.87 (8)	F1^viii^—Ca2—F7^xi^	115.01 (6)
F2—Zr1—F3	76.47 (9)	F1^vi^—Ca2—F7^xi^	58.94 (6)
F8—Zr1—F3	114.32 (10)	F6^ix^—Ca2—F7^xi^	60.56 (6)
F6—Zr1—F3	76.16 (8)	F6^x^—Ca2—F7^xi^	143.78 (7)
F10—Zr1—F3	141.78 (8)	F2^xi^—Ca2—F7^xi^	51.62 (6)
F7—Zr1—F11	143.54 (8)	F2—Ca2—F7^xi^	126.67 (6)
F2—Zr1—F11	141.23 (9)	F4—Ca2—F7^xi^	103.35 (6)
F8—Zr1—F11	76.60 (8)	F4^xi^—Ca2—F7^xi^	53.12 (6)
F6—Zr1—F11	74.05 (8)	F10^x^—Ca2—F7^xi^	100.25 (5)
F10—Zr1—F11	78.86 (8)	F10^ix^—Ca2—F7^xi^	97.57 (5)
F3—Zr1—F11	71.33 (9)	F7—Ca2—F7^xi^	155.57 (8)
F7—Zr1—F4	74.98 (8)	F8^i^—Ca3—F8^xii^	87.10 (12)
F2—Zr1—F4	72.62 (8)	F8^i^—Ca3—F3^vi^	156.41 (7)
F8—Zr1—F4	71.74 (8)	F8^xii^—Ca3—F3^vi^	98.78 (8)
F6—Zr1—F4	142.56 (8)	F8^i^—Ca3—F3	98.78 (8)
F10—Zr1—F4	141.80 (7)	F8^xii^—Ca3—F3	156.41 (7)
F3—Zr1—F4	75.04 (7)	F3^vi^—Ca3—F3	84.96 (13)
F11—Zr1—F4	117.61 (9)	F8^i^—Ca3—F9	84.10 (7)
F5^i^—Zr2—F5^ii^	143.17 (11)	F8^xii^—Ca3—F9	71.48 (7)
F5^i^—Zr2—F9^iii^	117.63 (7)	F3^vi^—Ca3—F9	76.31 (7)
F5^ii^—Zr2—F9^iii^	75.52 (7)	F3—Ca3—F9	131.65 (7)
F5^i^—Zr2—F9^iv^	75.52 (7)	F8^i^—Ca3—F9^vi^	71.48 (7)
F5^ii^—Zr2—F9^iv^	117.63 (7)	F8^xii^—Ca3—F9^vi^	84.10 (7)
F9^iii^—Zr2—F9^iv^	140.46 (11)	F3^vi^—Ca3—F9^vi^	131.65 (7)
F5^i^—Zr2—F11	140.20 (8)	F3—Ca3—F9^vi^	76.31 (7)
F5^ii^—Zr2—F11	74.04 (8)	F9—Ca3—F9^vi^	146.27 (10)
F9^iii^—Zr2—F11	77.57 (8)	F8^i^—Ca3—F5	132.51 (7)
F9^iv^—Zr2—F11	71.76 (8)	F8^xii^—Ca3—F5	73.82 (7)
F5^i^—Zr2—F11^v^	74.04 (8)	F3^vi^—Ca3—F5	70.82 (6)
F5^ii^—Zr2—F11^v^	140.20 (8)	F3—Ca3—F5	85.66 (7)
F9^iii^—Zr2—F11^v^	71.76 (8)	F9—Ca3—F5	126.99 (6)
F9^iv^—Zr2—F11^v^	77.57 (8)	F9^vi^—Ca3—F5	63.72 (6)
F11—Zr2—F11^v^	77.30 (13)	F8^i^—Ca3—F5^vi^	73.82 (7)
F5^i^—Zr2—F4^ii^	73.35 (7)	F8^xii^—Ca3—F5^vi^	132.51 (7)
F5^ii^—Zr2—F4^ii^	77.41 (8)	F3^vi^—Ca3—F5^vi^	85.66 (7)
F9^iii^—Zr2—F4^ii^	76.44 (8)	F3—Ca3—F5^vi^	70.82 (6)
F9^iv^—Zr2—F4^ii^	140.78 (7)	F9—Ca3—F5^vi^	63.72 (6)
F11—Zr2—F4^ii^	145.25 (7)	F9^vi^—Ca3—F5^vi^	126.99 (6)
F11^v^—Zr2—F4^ii^	115.20 (9)	F5—Ca3—F5^vi^	148.20 (10)
F5^i^—Zr2—F4^i^	77.41 (8)	F1—Ca4—F1^vi^	137.12 (11)
F5^ii^—Zr2—F4^i^	73.35 (7)	F1—Ca4—F2^vi^	69.36 (7)
F9^iii^—Zr2—F4^i^	140.78 (7)	F1^vi^—Ca4—F2^vi^	102.12 (7)
F9^iv^—Zr2—F4^i^	76.44 (8)	F1—Ca4—F2	102.12 (7)
F11—Zr2—F4^i^	115.20 (9)	F1^vi^—Ca4—F2	69.36 (7)
F11^v^—Zr2—F4^i^	145.25 (7)	F2^vi^—Ca4—F2	157.52 (10)
F4^ii^—Zr2—F4^i^	73.99 (11)	F1—Ca4—F7^ix^	129.24 (8)
F1—Ca1—F5^vi^	78.04 (7)	F1^vi^—Ca4—F7^ix^	78.34 (7)
F1—Ca1—F6	81.27 (8)	F2^vi^—Ca4—F7^ix^	67.84 (6)
F5^vi^—Ca1—F6	127.77 (7)	F2—Ca4—F7^ix^	127.36 (7)
F1—Ca1—F7^vii^	73.44 (8)	F1—Ca4—F7^xiii^	78.34 (7)
F5^vi^—Ca1—F7^vii^	106.33 (7)	F1^vi^—Ca4—F7^xiii^	129.24 (8)
F6—Ca1—F7^vii^	112.73 (7)	F2^vi^—Ca4—F7^xiii^	127.36 (7)
F1—Ca1—F10^v^	121.56 (7)	F2—Ca4—F7^xiii^	67.84 (6)
F5^vi^—Ca1—F10^v^	155.59 (7)	F7^ix^—Ca4—F7^xiii^	107.84 (10)
F6—Ca1—F10^v^	73.18 (7)	F1—Ca4—F10^ix^	144.10 (7)
F7^vii^—Ca1—F10^v^	69.93 (7)	F1^vi^—Ca4—F10^ix^	75.15 (7)
F1—Ca1—F9^iv^	154.42 (7)	F2^vi^—Ca4—F10^ix^	127.81 (7)
F5^vi^—Ca1—F9^iv^	77.06 (7)	F2—Ca4—F10^ix^	71.47 (6)
F6—Ca1—F9^iv^	109.85 (7)	F7^ix^—Ca4—F10^ix^	60.51 (6)
F7^vii^—Ca1—F9^iv^	119.50 (7)	F7^xiii^—Ca4—F10^ix^	66.43 (7)
F10^v^—Ca1—F9^iv^	84.00 (7)	F1—Ca4—F10^xiii^	75.15 (7)
F1—Ca1—F3	72.14 (9)	F1^vi^—Ca4—F10^xiii^	144.10 (7)
F5^vi^—Ca1—F3	66.37 (7)	F2^vi^—Ca4—F10^xiii^	71.47 (6)
F6—Ca1—F3	61.73 (7)	F2—Ca4—F10^xiii^	127.81 (7)
F7^vii^—Ca1—F3	145.59 (7)	F7^ix^—Ca4—F10^xiii^	66.43 (7)
F10^v^—Ca1—F3	130.46 (7)	F7^xiii^—Ca4—F10^xiii^	60.51 (6)
F9^iv^—Ca1—F3	92.47 (8)	F10^ix^—Ca4—F10^xiii^	81.52 (9)
F1—Ca1—F8^vii^	116.23 (8)	F1—Ca4—F3	63.80 (7)
F5^vi^—Ca1—F8^vii^	77.68 (7)	F1^vi^—Ca4—F3	79.01 (6)
F6—Ca1—F8^vii^	153.18 (7)	F2^vi^—Ca4—F3	104.03 (7)
F7^vii^—Ca1—F8^vii^	58.87 (7)	F2—Ca4—F3	54.59 (6)
F10^v^—Ca1—F8^vii^	80.17 (7)	F7^ix^—Ca4—F3	153.52 (7)
F9^iv^—Ca1—F8^vii^	63.55 (6)	F7^xiii^—Ca4—F3	97.08 (6)
F3—Ca1—F8^vii^	140.74 (8)	F10^ix^—Ca4—F3	125.49 (6)
F1—Ca1—F11	121.92 (8)	F10^xiii^—Ca4—F3	136.83 (6)
F5^vi^—Ca1—F11	95.13 (7)	F1—Ca4—F3^vi^	79.01 (6)
F6—Ca1—F11	57.87 (7)	F1^vi^—Ca4—F3^vi^	63.80 (7)
F7^vii^—Ca1—F11	156.31 (7)	F2^vi^—Ca4—F3^vi^	54.59 (6)
F10^v^—Ca1—F11	86.38 (7)	F2—Ca4—F3^vi^	104.03 (7)
F9^iv^—Ca1—F11	55.22 (6)	F7^ix^—Ca4—F3^vi^	97.08 (6)
F3—Ca1—F11	53.29 (7)	F7^xiii^—Ca4—F3^vi^	153.52 (7)
F8^vii^—Ca1—F11	118.27 (6)	F10^ix^—Ca4—F3^vi^	136.83 (6)
F1^viii^—Ca2—F1^vi^	154.59 (11)	F10^xiii^—Ca4—F3^vi^	125.49 (6)
F1^viii^—Ca2—F6^ix^	122.41 (8)	F3—Ca4—F3^vi^	60.32 (8)
F1^vi^—Ca2—F6^ix^	77.99 (7)	Ca1—F1—Ca4	127.94 (11)
F1^viii^—Ca2—F6^x^	77.99 (7)	Ca1—F1—Ca2^vii^	121.63 (10)
F1^vi^—Ca2—F6^x^	122.41 (8)	Ca4—F1—Ca2^vii^	109.64 (7)
F6^ix^—Ca2—F6^x^	83.69 (10)	Zr1—F2—Ca4	126.50 (10)
F1^viii^—Ca2—F2^xi^	67.95 (7)	Zr1—F2—Ca2	114.89 (9)
F1^vi^—Ca2—F2^xi^	110.54 (6)	Ca4—F2—Ca2	101.62 (6)
F6^ix^—Ca2—F2^xi^	71.13 (7)	Zr1—F3—Ca3	142.87 (12)
F6^x^—Ca2—F2^xi^	114.00 (7)	Zr1—F3—Ca1	99.48 (8)
F1^viii^—Ca2—F2	110.54 (6)	Ca3—F3—Ca1	107.24 (8)
F1^vi^—Ca2—F2	67.95 (7)	Zr1—F3—Ca4	97.99 (9)
F6^ix^—Ca2—F2	114.00 (7)	Ca3—F3—Ca4	107.36 (8)
F6^x^—Ca2—F2	71.13 (7)	Ca1—F3—Ca4	89.55 (7)
F2^xi^—Ca2—F2	173.59 (9)	Zr2^iii^—F4—Zr1	143.70 (12)
F1^viii^—Ca2—F4	78.94 (7)	Zr2^iii^—F4—Ca2	113.76 (8)
F1^vi^—Ca2—F4	78.93 (7)	Zr1—F4—Ca2	102.38 (9)
F6^ix^—Ca2—F4	156.54 (6)	Zr2^iii^—F5—Ca1^vi^	134.66 (9)
F6^x^—Ca2—F4	112.50 (7)	Zr2^iii^—F5—Ca3	110.14 (8)
F2^xi^—Ca2—F4	113.82 (7)	Ca1^vi^—F5—Ca3	114.36 (8)
F2—Ca2—F4	59.90 (6)	Zr1—F6—Ca1	111.56 (9)
F1^viii^—Ca2—F4^xi^	78.93 (7)	Zr1—F6—Ca2^xiii^	125.08 (9)
F1^vi^—Ca2—F4^xi^	78.94 (7)	Ca1—F6—Ca2^xiii^	120.46 (8)
F6^ix^—Ca2—F4^xi^	112.50 (6)	Zr1—F7—Ca1^viii^	117.66 (9)
F6^x^—Ca2—F4^xi^	156.54 (6)	Zr1—F7—Ca4^x^	111.69 (8)
F2^xi^—Ca2—F4^xi^	59.90 (6)	Ca1^viii^—F7—Ca4^x^	110.63 (8)
F2—Ca2—F4^xi^	113.82 (7)	Zr1—F7—Ca2	92.24 (7)
F4—Ca2—F4^xi^	58.49 (9)	Ca1^viii^—F7—Ca2	92.03 (6)
F1^viii^—Ca2—F10^x^	64.60 (6)	Ca4^x^—F7—Ca2	131.65 (8)
F1^vi^—Ca2—F10^x^	138.65 (7)	Zr1—F8—Ca3^iii^	147.21 (10)
F6^ix^—Ca2—F10^x^	60.87 (6)	Zr1—F8—Ca1^viii^	101.38 (9)
F6^x^—Ca2—F10^x^	52.89 (6)	Ca3^iii^—F8—Ca1^viii^	105.84 (7)
F2^xi^—Ca2—F10^x^	61.59 (5)	Zr2^i^—F9—Ca3	110.58 (8)
F2—Ca2—F10^x^	123.90 (6)	Zr2^i^—F9—Ca1^xiv^	124.23 (9)
F4—Ca2—F10^x^	142.40 (5)	Ca3—F9—Ca1^xiv^	114.33 (8)
F4^xi^—Ca2—F10^x^	118.98 (5)	Zr1—F10—Ca1^v^	143.51 (9)
F1^viii^—Ca2—F10^ix^	138.65 (7)	Zr1—F10—Ca4^x^	107.27 (8)
F1^vi^—Ca2—F10^ix^	64.60 (6)	Ca1^v^—F10—Ca4^x^	106.96 (7)
F6^ix^—Ca2—F10^ix^	52.89 (6)	Zr1—F10—Ca2^xiii^	98.83 (7)
F6^x^—Ca2—F10^ix^	60.87 (6)	Ca1^v^—F10—Ca2^xiii^	97.15 (6)
F2^xi^—Ca2—F10^ix^	123.90 (6)	Ca4^x^—F10—Ca2^xiii^	83.57 (6)
F2—Ca2—F10^ix^	61.59 (5)	Zr2—F11—Zr1	161.30 (11)
F4—Ca2—F10^ix^	118.98 (5)	Zr2—F11—Ca1	105.85 (7)
F4^xi^—Ca2—F10^ix^	142.40 (5)	Zr1—F11—Ca1	92.79 (8)
F10^x^—Ca2—F10^ix^	85.95 (7)		

Symmetry codes: (i) −*x*+3/2, *y*−1/2, −*z*+1; (ii) *x*−1/2, −*y*+1/2, −*z*+1; (iii) −*x*+3/2, *y*+1/2, −*z*+1; (iv) *x*−1/2, −*y*−1/2, −*z*+1; (v) −*x*+1, −*y*, *z*; (vi) −*x*+2, −*y*, *z*; (vii) *x*, *y*−1, *z*; (viii) *x*, *y*+1, *z*; (ix) *x*+1/2, −*y*+1/2, −*z*+2; (x) −*x*+3/2, *y*+1/2, −*z*+2; (xi) −*x*+2, −*y*+1, *z*; (xii) *x*+1/2, −*y*+1/2, −*z*+1; (xiii) −*x*+3/2, *y*−1/2, −*z*+2; (xiv) *x*+1/2, −*y*−1/2, −*z*+1.
